# How measurement science can improve confidence in research results

**DOI:** 10.1371/journal.pbio.2004299

**Published:** 2018-04-23

**Authors:** Anne L. Plant, Chandler A. Becker, Robert J. Hanisch, Ronald F. Boisvert, Antonio M. Possolo, John T. Elliott

**Affiliations:** 1 Material Measurement Laboratory, National Institute of Standards and Technology, Gaithersburg, Maryland, United States of America; 2 Information Technology Laboratory, National Institute of Standards and Technology, Gaithersburg, Maryland, United States of America

## Abstract

The current push for rigor and reproducibility is driven by a desire for confidence in research results. Here, we suggest a framework for a systematic process, based on consensus principles of measurement science, to guide researchers and reviewers in assessing, documenting, and mitigating the sources of uncertainty in a study. All study results have associated ambiguities that are not always clarified by simply establishing reproducibility. By explicitly considering sources of uncertainty, noting aspects of the experimental system that are difficult to characterize quantitatively, and proposing alternative interpretations, the researcher provides information that enhances comparability and reproducibility.

## Indicators of confidence in research results

While reports about the difficulty of reproducing published biomedical research results in the labs of pharmaceutical companies [1,2] have in large part triggered the current “reproducibility crisis,” reproducibility has also been cited as a concern in computation [3], forensics [4], epidemiology [5], psychology [6], and other fields, including chemistry, biology, physics and engineering, medicine, and earth and environmental sciences [7].

While “reproducibility” is the term most often used to describe the issue, it has been frequently pointed out that reproducibility does not guarantee that a result of scientific inquiry tracks the truth [8–11]. It has been suggested that, instead, there is a need for “a fundamental embrace of good scientific methodology” [12], and the term “metascience” has been proposed to refer to the idea that rigorous methods can be used to examine the reliability of results [13].

These perspectives suggest that it would be worthwhile to consider how the concepts of measurement science—i.e., metrology—can provide useful guidance that would enable researchers to assess and achieve rigor of a research study [14]. The goal of measurement science is comparability, which enables evaluation of the results from one time and place relative to results from another time and place; this is ultimately the goal of establishing rigor and reproducibility. The purpose of this manuscript is to provide a practical connection between the field of metrology and the desire for rigor and reproducibility in scientific studies.

In the field of metrology, a measurement consists of two components: a value determined for the measurand and the uncertainty in that value [15]. The uncertainty around a value is an essential component of a measurement. In the simplest case, the uncertainty is determined by the variability in replicate measurements, but for complicated measurements, it is estimated by the combination of the uncertainties at every step in the process. The concepts that support quantifying measurement uncertainty arise from international conventions that have been agreed to through consensus by scientists in many fields of study over the past 150 years and continue to be developed. These conventions are developed and adopted by the National Metrology Institutes around the world (including the National Institute of Standards and Technology [NIST] in the United States) and international standards organizations such as the International Bureau of Weights and Measures (Bureau International des Poids et Mesures, BIPM), the International Electrotechnical Commission (IEC), the International Federation of Clinical Chemistry and Laboratory Medicine (IFCC), the International Organization for Standardization (ISO), the International Union of Pure and Applied Physics (IUPAP), the International Laboratory Accreditation Cooperation (ILAC), and others. These efforts helped to advance the concepts of modern physics by providing the basis on which comparison of data was made possible [14]. Thus, it seems appropriate to examine these concepts today to inform our current concerns about rigor and reproducibility.

One of the consensus documents developed by measurement scientists is the *Guide to Expression of Uncertainty in Measurement* [16], commonly known as the GUM. This document describes the types of uncertainty (e.g., Type A, those that are evaluated by statistical methods; and Type B, those that are evaluated by other means) and methods for evaluating and expressing uncertainties. The GUM describes a rigorous approach to quantifying measurement uncertainty that is more readily applied to well-defined physical quantities with discrete values and uncertainties (such as the measurements of amount of a substance, like lead in water) than to measurements that involve many parameters (such as complex experimental studies involving cells and animals). Calculating uncertainties in such complex measurement systems is a topic of ongoing research. But even if uncertainties are not rigorously quantified, the concepts of measurement uncertainty provide a systematic thought process about to how to critically evaluate comparability between results produced in different laboratories.

The GUM identifies examples of sources of uncertainty. These include an incomplete definition of what is being measured (i.e., the measurand); the possibility of nonrepresentative or incomplete sampling, in which the samples measured may not represent all of what was intended to be measured; the approximations and assumptions that are incorporated in the measurement method and procedure; and inadequate knowledge of the effects of environmental conditions on the measurement. In Table 1, we have grouped the sources of uncertainty identified in the GUM that are common to many scientific studies, and we have indicated measurement science approaches for characterizing and mitigating uncertainty.

**Table 1:** Identifying, reporting, and mitigating sources of uncertainty in a research study.

**1. State the plan** a. Clearly articulate the goals of the study and the basis for generalizability to other settings, species, conditions, etc., if claimed in the conclusions. b. State the experimental design, including variables to be tested, numbers of samples, statistical models to be used, how sampling is performed, etc. c. Provide preliminary data or evaluations that support the selection of protocols and statistical models. d. Identify and evaluate assumptions related to anticipated experiments, theories, and methods for analyzing results.
**2. Look for systemic sources of bias and uncertainty** a. Characterize reagents and control samples (e.g., composition, purity, activity, etc.). b. Ensure that experimental equipment is responding correctly (e.g., through use of calibration materials and verification of vendor specifications). c. Show that positive and negative control samples are appropriate in composition, sensitivity, and other characteristics to be meaningful indictors of the variables being tested. d. Evaluate the experimental environment (e.g., laboratory conditions such as temperature and temperature fluctuations, humidity, vibration, electronic noise, etc.).
**3. Characterize the quality and robustness of experimental data and protocols** a. Acquire supplementary data that provide indicators of the quality of experimental data. These indicators include precision (i.e., repeatability, with statistics such as standard deviation and variance), accuracy (which can be assessed by applying alternative [orthogonal] methods or by comparison to a reference material), sensitivity to environmental or experimental perturbants (by testing for assay robustness to putatively insignificant experimental protocol changes), and the dynamic range and response function of the experimental protocol or assay (and assuring that data points are within that valid range). b. Reproduce the data using different technicians, laboratories, instruments, methods, etc. (i.e., meet the conditions for reproducibility as defined in the VIM).
**4. Minimize bias in data reduction and interpretation of results** a. Justify the basis for the selected statistical analyses. b. Quantify the combined uncertainties of the values measured using methods in the GUM [16] and other sources [17]. c. Evaluate the robustness and accuracy of algorithms, code, software, and analytical models to be used in analysis of data (e.g., by testing against reference datasets). d. Compare data and results with previous data and results (yours and others’). e. Identify other uncontrolled potential sources of bias or uncertainty in the data. f. Consider feasible alternative interpretations of the data. g. Evaluate the predictive power of models used.
**5. Minimize confusion and uncertainty in reporting and dissemination** a. Make available all supplementary material that fully describes the experiment/simulation and its analysis. b. Release well-documented data and code used in the study. c. Collect and archive metadata that provide documentation related to process details, reagents, and other variables; include with numerical data as part of the dataset.

**Abbreviations**: GUM, *Guide to Expression of Uncertainty in Measurement*; VIM, *International Vocabulary of Basic and General Terms in Metrology*

The GUM also provides definitions of many terms such as “repeatability” (which is defined as the closeness of the agreement between the results of successive measurements of the same measurand carried out under the same conditions of measurement) and “reproducibility” (which is defined as the closeness of the agreement between the results of measurements of the same measurand carried out under different conditions of measurement). A complete list of consensus definitions of measurement-related terms can be found in the *International Vocabulary of Basic and General Terms in Metrology* (VIM) [18]. A recent publication demonstrates the adoption of these definitions to harmonize practices across the geophysics community [19].

## What does Table 1 add to existing efforts?

There have been many efforts to encourage more reliable research results, and many fields have proposed or instituted conventions, checklists, requirements, and reporting standards that are applicable to their specific disciplines. Some of these include the Grades of Recommendation, Assessment, Development and Evaluation (GRADE) approach for assessing clinical evidence [20], the minimum information activities that have a long history in the biosciences (e.g., Minimum Information about a Microarray Experiment [MIAME]) [21], checklists developed by scientific journals requiring specific criteria to be reported [22], a NIST system for checking thermodynamic data prior to publication [23], and many more. These efforts are not intended to be comprehensive determinations of potential sources of uncertainty in measurement. But interest in measurement science principles is increasing. For example, the Minimum Information About a Cellular Assay (MIACA) activity [24], which was last updated in 2013, encourages reporting the experimental details of cellular assay projects. The more recent Minimum Information About T cell Assays (MIATA), [25,26] which is focused on identifying and encouraging the reporting of variables of particular importance to the outcome of T cell assays, is more comprehensive. MIATA guidelines go beyond descriptions of activities and reagents to include the reporting of quality control activities such as providing information regarding the strategies for data analysis and reporting any effort to pretest medium or serum for assay performance. The most current National Institutes of Health (NIH) instructions for grant applications [27] speak to many of the concepts of metrology: stating the scientific premise and considering the strengths and weaknesses of prior research; applying scientific method to experimental design, methodology, analysis, and interpretation; considering biological variables such as sex; and authenticating biological and chemical resources that may be sources of variability. Thus, it seems timely to suggest a comprehensive framework that can help to guide identification of the many other potential sources of uncertainty. The conceptual framework in Table 1 can enhance existing guidelines by helping scientists identify potential sources of uncertainty that might not have been considered in existing checklists and to provide some strategies for reducing uncertainty. Table 1 is designed to help guide researchers’ critical thinking about the various aspects of their research in an organized way that encourages them to document the data they can, and often do, collect that provide confidence in the results.

The inclusion of supporting evidence helps end users of research results—such as decision-makers, commercial developers, and other researchers—know how best to use and follow up on the results. Few research studies will address all aspects indicated in Table 1. But by explicitly acknowledging what is known—or, more importantly, what isn’t known—about the various components of a research effort, it is easier to see the strengths and limitations of a study and to assess, for example, whether the study is more preliminary in nature or if the results are highly reliable. The Data Readiness Level is a concept that has been put forward by the nanotechnology community and is an example of this kind of approach, [28] and others have suggested the need for this level of reporting [11].

## What are the hurdles that keep ideas such as these from being implemented?

The sociological issues that accompany the “reproducibility crisis” have been discussed in many venues and are beyond the scope of this discussion. Instead, we focus on the principles and practices of measurement science since we find that researchers, particularly in rapidly advancing fields, are sometimes confused about how to apply these principles of the scientific method to achieve “rigor and reproducibility.”

A hurdle to implementation of these concepts is the need for tools and technologies that can reduce the challenges for experimentalists who want to address the elements in Table 1. There has not been sufficient investment, perhaps, in technologies that could allow us to better characterize the components of our experimental systems, such as antibody reagents, cell lines, or image analysis pipelines. As a scientific community, we have not prioritized investments in software to facilitate collecting information on complex experimental protocols. While there is great interest in data mining, there is still a lack of progress in the development of natural language and other approaches for achieving harmonized vocabularies that would make it easier to compare and share experimental metadata and protocols. Efforts associated with capturing the details of complicated experimental protocols are being undertaken. PLOS has entered into a collaboration with Protocols.io [29] to facilitate reporting, sharing, and improving protocols. Another effort, ProtocolNavigator [30], enables collection of highly detailed experimental information and storage of provenance information; there are also supporting links to stored data and explanatory videos [31]. Challenges associated with data and digital resources are being considered by the Research Data Alliance (RDA) [32]. The RDA was established in 2013 to foster the sharing of research data but recognized that effective sharing requires standards and best practices and is pursuing technical developments in data discovery, semantics, ontologies, data citation and versioning, data types, and persistent identifiers. Also, with the current emphasis on open data [33] and large-scale data sharing [32], it would be helpful to have a means of evaluating the aspects of the research that establish confidence of the results being shared, especially by those who are using data outside of their area of technical expertise. In addition, increased support for the science that underpins the technologies and methods that help to establish confidence in data will contribute to improving the reusability of published research results.

## Conclusions

The consideration by researchers of a systematic approach to identifying sources of uncertainty will enhance comparability of results between laboratories. Because no single scientific observation reveals the absolute “truth,” the job of the researcher and the reviewer is to determine how ambiguities have been reduced and what ambiguities still exist. By addressing and characterizing the components of the study as potential sources of uncertainty, the researcher can provide the supporting evidence that helps to define the characteristics of the data, analysis, and tests of the assumptions that were made; such evidence provides confidence in the results and helps inform the reader about how to use the information. Unfortunately, even when studies include these activities, they are rarely reported in an explicit and systematic way that provides maximum value to the reader.

A framework such as the one outlined in Table 1 is applicable to many areas of scientific research. The ideas presented here are not radical or new but are worthy of reconsideration because of the current concern about comparability of research results. We provide this information in the spirit of stimulating discussion within and among the scientific disciplines. More explicit use and documentation of the concepts discussed above will improve confidence in published research results. Applying these concepts will require commitment and critical thinking on the part of individuals, as well as a continuation of the tradition of cooperative effort within and across scientific communities. The end result will be worth the additional effort.

## Abbreviations

BIPM: International Bureau of Weights and Measures (Bureau International des Poids et Mesures)
GRADE: Grades of Recommendation, Assessment, Development and Evaluation
GUM: *Guide to Expression of Uncertainty in Measurement*
IEC: International Electrotechnical Commission
IFCC: International Federation of Clinical Chemistry and Laboratory Medicine
ILAC: International Laboratory Accreditation Cooperation
ISO: International Organization for Standardization
IUPAP: International Union of Pure and Applied Physics
MIACA: Minimum Information About a Cellular Assay
MIAME: Minimum Information about a Microarray Experiment
MIATA: Minimum Information About T-cell Assays
NIH: National Institutes of Health
NIST: National Institute of Standards and Technology
RDA: Research Data Alliance
VIM: *International Vocabulary of Basic and General Terms in Metrology*
